# Interaction effects of *GIT1* and *DRD4* gene variants on continuous performance test variables in patients with ADHD

**DOI:** 10.1002/brb3.785

**Published:** 2017-08-01

**Authors:** Hyojin Kim, Johanna Inhyang Kim, Haebin Kim, Jae‐Won Kim, Bung‐Nyun Kim

**Affiliations:** ^1^ Division of Child and Adolescent Psychiatry Department of Psychiatry Seoul National University College of Medicine Seoul Republic of Korea; ^2^ Department of Public Health Medical Services Seoul National University Bundang Hospital Seong‐nam Gyeonggi‐do Republic of Korea

**Keywords:** attention deficit hyperactivity disorder, continuous performance test, DRD4 gene, endophenotypes, gene–gene interaction, *GIT1* gene, impulsivity, neuropsychological test, synapses

## Abstract

**Introduction:**

The G protein‐coupled receptor kinase interacting protein 1 gene (*GIT1*) has been proposed to be a risk gene for attention deficit hyperactivity disorder (ADHD), and it regulates the endocytosis of G protein‐coupled receptors like dopamine receptors. The purpose of this study was to investigate the interaction effects of *GIT1* and dopamine receptor D4 (*DRD4*) gene variants on variables of the continuous performance test (CPT).

**Methods:**

This study recruited 255 ADHD patients and 98 healthy controls (HC) who underwent CPT and genetic analyses. The genotypes were classified into two groups (the C/C and C/T genotype groups for *GIT1,* 4R homozygotes and others for *DRD4*) and the genotype × genotype effects were examined using hierarchical multivariable linear regression analyses.

**Results:**

There were significant *GIT1 *× *DRD4* effects for commission errors on the CPT in the ADHD group (*p* = .006). In contrast, there were no significant *GIT1 *× *DRD4* effects on any CPT variables in the HC.

**Conclusions:**

The present findings demonstrated that there were significant interaction effects of the *GIT1* and *DRD4* gene variants on impulsivity in ADHD. Replication studies with larger sample sizes that include patients from various ethnic backgrounds are warranted to confirm these findings.

## 1 INTRODUCTION

Attention deficit hyperactivity disorder (ADHD) is a neurodevelopmental disorder characterized by developmentally inappropriate levels of inattention, hyperactivity, and/or impulsivity (American Psychiatric Association, 2013). Family, twin, and adoption studies of ADHD patients have estimated its heritability to be approximately 75% (Faraone et al., 2005), and the findings of genetic studies indicate that ADHD is a complex polygenic disorder. Although a majority of studies investigating ADHD have focused on catecholamine dysregulation and dopamine‐related genes (e.g., dopamine receptor D4 [*DRD4*]) that are related to attentional processes (Gizer, Ficks, & Waldman, 2009), copy number variation and genome‐wide association studies (GWAS) have identified several neurodevelopmental genes as possible candidate genes involved in ADHD (Li, Chang, Zhang, Gao, & Wang, 2014; Poelmans, Pauls, Buitelaar, & Franke, 2011).

The G protein‐coupled receptor kinase interacting protein 1 (*GIT1*) gene, which is located on chromosome 17p11.2, has been suggested as a novel candidate gene for ADHD (Won et al., 2011). *GIT1* is the gene for a multifunctional adaptor protein and plays an important role in cell migration (Penela, Nogues, & Mayor, 2014; Podufall et al., 2014) and neurite outgrowth (Li et al., 2016). Furthermore, *GIT1* regulates synapse formation (Kim et al., 2003; Menon et al., 2010; Zhang, Webb, Asmussen, & Horwitz, 2003) and the endocytosis of β_2_‐adrenergic receptors and other G protein‐coupled receptors (Claing et al., 2000; Premont et al., 1998). Won et al. (2011) evaluated 27 single nucleotide polymorphisms (SNPs) in the *GIT1* gene and found that rs550818 is associated with the risk of ADHD in Korean children. However, a Brazilian case–control study and a recent meta‐analysis could not replicate this finding (Klein et al., 2015; Salatino‐Oliveira et al., 2012); this discrepancy may be due to the fact that these studies primarily included subjects with Caucasian backgrounds. As different genetic backgrounds are associated with different allele frequencies and risks for diseases, the interpretation of these findings is difficult.

To determine the relationship of *GIT1* with ADHD, studies investigating the interaction of *GIT1* with other candidate ADHD genes may be helpful. Of the genes that are known to be associated with ADHD, the *DRD4* exon III 48 base pair variable number tandem repeats (VNTR) polymorphism is one of the most extensively investigated candidates. DRD4 proteins are expressed in multiple brain regions that are thought to be involved in the etiology of ADHD (Floresco & Tse, 2007; Noain et al., 2006). The *DRD4* gene is located on chromosome 11p15.5; the exon III 48 bp VNTR polymorphism can include 2–11 repeats. Recent meta‐analyses have suggested that the *DRD4* 7‐repeat (7R) allele is associated with ADHD (Faraone & Mick, 2010; Li, Sham, Owen, & He, 2006); however, Asian populations, including Koreans, rarely exhibit this allele (Tomitaka et al., 1999). Instead, in the Korean population, the 4‐repeat (4R) allele has been found to be associated with variables on the continuous performance test (CPT) and methylphenidate treatment response (Cheon, Kim, & Cho, 2007; Kim et al., 2009).

As *GIT1* plays an important role in regulation of the endocytic traffic of numerous G protein‐coupled receptors, including dopamine receptors (Claing et al., 2000; Premont et al., 1998), it is possible that the actions of *GIT1* and *DRD4* are influenced by each other. It is also known that specific endophenotypes tend to be advantageous in terms of statistical power in genetic studies with small sample sizes and may provide insight into how genetic variants affect behavioral phenotypes (Durston, 2010). Therefore, this study utilized variables associated with the CPT as endophenotypes of ADHD. The primary purpose of this study was to investigate the independent and interaction effects of *GIT1* rs550818 and the *DRD4* 48‐bp VNTR 4R allele on CPT variables in patients with ADHD. As *GIT1*‐/‐ mice exhibit hyperactivity and impaired learning and memory (Won et al., 2011) and the *DRD4* 4R allele is associated with commission errors in the Korean population (Kim et al., 2009), it was hypothesized that there would be significant interaction effects between *GIT1* and *DRD4* on the CPT variables.

## 2 MATERIALS AND METHODS

### 2.1 Participants

This study included 255 children and adolescents with ADHD and 98 healthy controls (HC) between 6 and 17 years of age who were recruited between August 2010 and February 2015. For this study, participants from two studies that were conducted using the same protocol were combined into a single subject pool; detailed explanations of both study protocols and the combined protocol have been provided elsewhere (Kim et al., 2016). The first study initially recruited 90 ADHD patients and 33 HCs; after excluding five ADHD patients with missing CPT data and one HC with missing genetic data, 85 ADHD patients and 32 HCs were assessed (Hong et al., 2015). The second study initially recruited 191 ADHD patients and 78 HCs; after excluding four patients with missing visual data and 17 patients with missing genetic data from the ADHD group and one subject with missing CPT data and 11 subjects with missing genetic data from the HC group, 170 ADHD patients and 66 HCs were assessed (Park et al., 2015).

All of the ADHD patients were medication naïve, of Korean ethnicity, and had visited the Child and Adolescent Psychiatry outpatient clinic at the Seoul National University Hospital. ADHD and other psychiatric comorbidities were confirmed according to the criteria of the Diagnostic and Statistical Manual of Mental Disorders, Fourth Edition (DSM‐IV) by board‐certified child and adolescent psychiatrists using the Kiddie Schedule for Affective Disorders and Schizophrenia‐Present and Lifetime version (K‐SADS‐PL) (Kaufman et al., 1997; Kim et al., 2004). The exclusion criteria for ADHD were as follows: IQ < 70; a hereditary genetic disorder; current or past history of brain trauma, organic brain disorder, seizure, or any neurological disorder; autism spectrum disorder, communication disorder, or learning disorder; schizophrenia or any other childhood‐onset psychotic disorder; major depressive disorder or bipolar disorder; Tourette's syndrome or a chronic motor/vocal tic disorder; obsessive compulsive disorder; and/or a history of methylphenidate treatment lasting for more than 1 year or having taken the drug within the past 4 weeks. The HC group included typical‐development children and adolescents who were free of any psychiatric diagnoses according to the K‐SADS‐PL. The same exclusion criteria for the ADHD group were applied to the HC group with the additional criterion of an ADHD diagnosis.

IQ was measured using the Korean Educational Developmental Institute's Wechsler Intelligence Scale for Children (Park, Yoon, Park, & Kwon, 1996), and the severity of ADHD symptoms was measured using the parent‐completed ADHD Rating Scale‐IV (ADHD‐RS) (So, Noh, Kim, Ko, & Koh, 2002). Written informed consent was obtained from all parents/guardians and adolescents, and the children provided verbal assent to participate after sufficient explanation of the study prior to enrollment. All study protocols were approved by the Institutional Review Board of Seoul National University Hospital.

### 2.2 Genotyping

Genomic DNA was extracted from blood samples that had been frozen and stored using a G‐DEX II Genomic DNA Extraction Kit (Intron Biotechnology; Seongnam, Korea). SNPs were detected using Sanger Sequencing. For each SNP, a polymerase chain reaction (PCR) amplification was performed in 20 μl reactions with 1× PCR buffer, 200 μmol/L of deoxynucleotide triphosphates (dNTPs), 0.5 μmol/L forward and reverse primers (5′ AGCTGCTTGGCAGCCTTG and 5′‐ACC TGG GTG GAG ACA CAG AC‐3′ for rs550818 [*GIT1*]), 100 ng of gDNA, and 0.5 U Taq polymerase (Nanohelix; Daejeon, Korea). The reaction mixture procedure consisted of incubation at 95°C for 15 min followed by 40 cycles of 95°C for 60 s, 55°C for 30 s, and 72°C for 30 s with a final extension at 72°C for 5 min. Following the PCR procedure, unincorporated dNTPs and primers were removed using ExoStar 1 (GE Healthcare; Seongnam, Korea) with incubation at 37°C for 60 min followed by incubation at 85°C for 15 min for enzyme inactivation. The PCR products were directly sequenced using Big Dye Termination cycle sequencing (Applied Biosystems’; Foster City, CA) and the SNPs were manually determined via visual inspection.

To analyze the VNTR of *DRD4*, PCR amplification was performed in 20 μl reactions with 1× Pfu buffer; 200 μmol/L of deoxyadenosine triphosphate (dATP), deoxythymidine triphosphate (dTTP), and deoxycytidine triphosphate (dCTP); 100 μmol/L of deoxyguanosine triphosphate (dGTP), 100 μmol/L of 7‐deaza‐dGTP (Roche’; Penzberg, Germany), 0.5 μmol/L forward and reverse primers (5′‐ACC ACC ACC GGC AGG ACC CTC ATG GCC TTG CGC TC and 5′‐CTT CCT ACC CTG CCC GCT CAT GCT GCTGCT CTA CTG G‐3′), 1× Band Doctor (Solgent; Daejeon, Korea), 100 ng of gDNA, and 2 U Pfu Taq polymerase (Solgent). The reaction mixture procedure consisted of incubation at 98°C for 5 min followed by 35 cycles at 98°C for 45 s, 57°C for 45 s, and 72°C for 60 s with a final extension at 72°C for 10 min. After amplification, the PCR products were resolved in 2% agarose gel.

### 2.3 Continuous performance test

Impulsivity and inattention were measured using a Korean version of the computerized CPT that has well‐established validity and reliability (Shin, Cho, Chun, & Hong, 2000). Visual stimuli were presented on a screen for 100 ms every 2 s. The participants were instructed to respond to the target stimulus (square containing a triangle) but not to the non‐target stimuli (square containing either a square or a circle). Performance was assessed based on three variables: (1) omission errors (failure to respond) as a measure of inattention, (2) commission errors (false response) as a measure of impulsivity, and (3) response time variability (the standard deviation [SD] of the response times of correct responses) as a measure of sustained attention. All data were automatically transformed into *T*‐scores adjusted for age relative to a normal population of 847 children between 5 and 15 years of age (Shin et al., 2000); lower *T*‐scores indicate better performance.

### 2.4 Statistical analysis

The Hardy–Weinberg equilibrium was calculated for each gene variant using the goodness‐of‐fit Chi‐square test. The genotypes of the *GIT1* variant were classified as C/C and C/T, and the genotypes of the *DRD4* variant were classified as 4R allele homozygotes and others. Demographic and clinical characteristics were compared between ADHD and HC groups and between genotype groups of the *GIT1* variant using independent *t*‐tests for continuous variables and Chi‐square or Fisher's exact tests for categorical variables. The genotypic and allelic distributions of the *GIT1* and *DRD4* variants were compared between ADHD and HC groups with Chi‐square tests.

The main genotype effects and the genotype × genotype effects on CPT variables were tested using hierarchical multivariable linear regression analyses in the combined ADHD and HC (ADHD + HC) group and in the ADHD and HC groups independently. Age and gender were included in Block 1, the *GIT1* and *DRD4* genotype groups were included in Block 2, and *GIT1 *×* DRD4* was included in Block 3; in case of the ADHD + HC group, age and gender were included in Block 1, the *GIT1* genotype, *DRD4* genotype, and diagnosis were included in Block 2, and *GIT1 *×* DRD4* was included in Block 3. A post hoc analysis to determine the direction of interaction was conducted by investigating the effects of the *DRD4* variant genotype groups on CPT variables in the *GIT1* C/C and *GIT1* C/T groups independently.

To determine the three‐way interaction effect of diagnosis, and *GIT1* genotype and *DRD4* genotype groups, we further conducted multivariable linear regression analyses with each CPT variables as independent variables, and the main effects, two‐way interactions and three‐way interactions as dependent variables (Schaafsma et al., 2017). Diagnosis, *GIT1* and *DRD4* genotype groups were placed in Block 1, the diagnosis × *GIT1*, diagnosis × *DRD4*, and *GIT1 *×* DRD4* in Block 2, and diagnosis × *GIT1 *×* DRD4* was included in Block 3.

All statistical analyses were performed using SPSS ver. 22.0 software (SPSS Inc.; Chicago, IL) (SPSS: RRID: SCR_002865); a two‐tailed *p*‐value < .008 (0.05/[2 gene variants × 3 CPT variables]) was considered to indicate statistical significance.

## 3 RESULTS

The demographic and clinical characteristics of the ADHD and HC groups are presented in Table 1. As significant differences in age and gender were observed between the ADHD and HC groups, age and gender were included as covariates in the CPT analyses. There were no significant differences in annual income or paternal and maternal educational levels between the two groups. The ADHD group had significantly higher ADHD‐RS total, inattention, and hyperactivity–impulsivity scores (*p*‐values < .001). Of the CPT variables, there was a significant diagnosis effect for mean omission errors, commission errors, and response time variability (*p*‐values < .001). There were no significant differences in demographic and clinical characteristics according to the genotype group of the *GIT1* or *DRD4* variant in the ADHD and HC groups.

**Table 1:** Demographic and clinical characteristics of the ADHD and HC groups

Characteristic	ADHD (*n* = 255)	HC (*n* = 98)	*p*‐value
Age (years), mean (SD)	9.0 (2.4)	10.4 (2.9)	<.001
Gender (male), *N* (%)	199 (78.0)	54 (55.1)	<.001
Intelligence quotient, mean (SD)	105.7 (14.8)	113.9 (12.5)	<.001
Yearly family income > $25,000, *N* (%)	160 (70.2)	58 (62.4)	.174
Paternal education, years, mean (SD)	14.8 (1.9)	14.6 (2.0)	.394
Maternal education, years, mean (SD)	14.5 (2.3)	14.3 (3.1)	.476
ADHD subtype, *N* (%)			
Predominantly inattentive	97 (38.0)		
Predominantly hyperactive–impulsive	18 (7.1)		
Combined	114 (44.7)		
NOS	26 (10.2)		
ADHD rating scale score, mean (SD)			
Total score	25.2 (10.9)	5.5 (5.5)	<.001
Inattention subscore	14.9 (5.7)	3.7 (3.9)	<.001
Hyperactivity–impulsivity subscore	10.3 (6.6)	1.8 (2.1)	<.001
CPT variables *T*‐scores, mean (SD)			
Omission errors	65.2 (20.3)	53.7 (14.8)	<.001
Commission errors	65.0 (19.4)	55.4 (15.1)	<.001
Response time variability	62.6 (18.6)	51.3 (14.4)	<.001

ADHD, attention deficit/hyperactivity disorder; HC, healthy control; SD, standard deviation; NOS, not otherwise specified; CPT, continuous performance test.

The genotypic and allelic distributions of the two polymorphisms are shown in Table S1 and number of participants in each genotype group is presented in Table S2. Each of the genotypes of the gene variants was in agreement with the values expected based on the Hardy–Weinberg equilibrium (*p *>* *.05); no differences were observed in the allelic or genotypic frequencies of the *GIT1* and *DRD4* variants between ADHD and HC groups.

**Figure 1:**
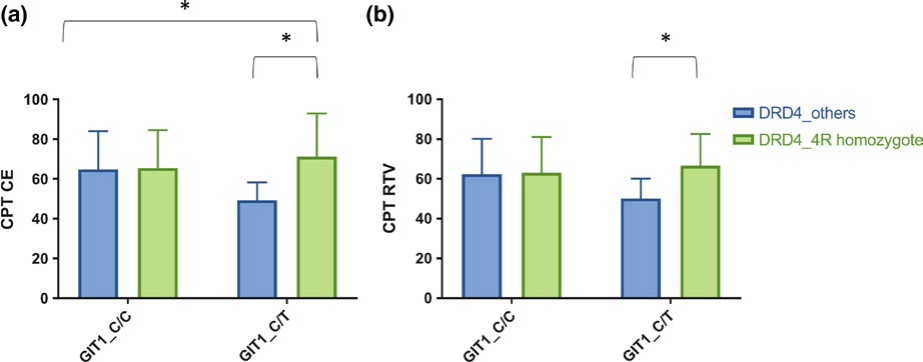
Interaction effects of *GIT1* and *DRD4* gene variants on continuous performance test (CPT) variables in ADHD. (a) Interaction effect of GIT1 and DRD4 on CPT commission errors (CE). (b) Interaction effect of GIT1 and DRD4 on CPT response time variability (RTV). *: *p* < .008

The genotype and genotype × genotype effects of the *GIT1* and *DRD4* variants on CPT are presented in Table 2. In the ADHD + HC group, there were significant effects of *DRD4* on commission errors (*p *=* *.003). In the ADHD group, there were significant effects of *DRD4* and *GIT1 *×* DRD4* on commission errors (*p *=* *.004, and *p *=* *.006, respectively, Figure 1). On the other hand, there were no main genotype effects of the *GIT1* or *DRD4* variants or an interaction effect of *GIT1 *×* DRD4* variants on any of the CPT variables in the HC group. The post hoc statistical power was 78%.

**Table 2:** Main and interaction effects of *GIT1* and *DRD4* genotypes on CPT variables in the ADHD and HC groups

Variables	Diagnosis		*GIT1*		*DRD4*		*GIT1 *×* DRD4* a		Cohen's *f* ^2^
	β (95% CI)	*p*‐value	β (95% CI)	*p*‐value	β (95% CI)	*p*‐value	β (95% CI)	*p*‐value	
CPT scores, mean (SD)									
ADHD + HC									
Omission errors	−7.46 (−11.95, −2.97)	.001	8.24 (−1.13, 17.61)	.085	8.20 (−2.74, 19.14)	.141	−7.15 (−18.83, 4.52)	.229	0.22
Commission errors	−5.45 (−9.80, −1.10)	.014	12.03 (2.95, 21.10)	.010	16.02 (5.42, 26.61)	.003	−15.23 (−26.53, −3.92)	.008	0.19
Response time variability	−8.66 (−12.76, −4.56)	.031	7.69 (−0.87, 16.24)	.078	12.00 (2.01, 21.99)	.019	11.74 (−22.40, −1.08)	.031	0.16
ADHD	NA								
Omission errors			14.03 (2.56, 25.49)	.017	13.91 (0.78, 27.03)	.038	−11.97 (−26.04, 2.11)	.095	0.17
Commission errors			13.88 (2.82, 24.95)	.014	18.95 (6.28, 31.61)	.004	−19.08 (−32.66, −5.50)	.006	0.15
Response time variability			11.35 (0.93, 21.76)	.025	14.90 (2.97, 26.82)	.015	−14.64 (−27.43, −1.86)	.025	0.07
HC	NA								
Omission errors			−9.76 (−24.92, 5.40)	.204	−8.57 (−27.85, 10.71)	.380	8.30 (−11.97, 28.56)	.418	0.11
Commission errors			4.54 (−10.66, 19.74)	.555	2.12 (−17.21, 21.46)	.828	1.63 (−18.69, 21.95)	.159	0.15
Response time variability			−4.53 (−19.04, 9.99)	.537	3.14 (−15.33, 21.60)	.737	−2.62 (−22.03, 16.79)	.789	0.14

CPT, continuous performance test; ADHD, attention deficit/hyperactivity disorder; HC, healthy control; ADHD + HC, ADHD and HC groups combined: CI, confidence interval; SD, standard deviation.

a Age and gender in first block, *GIT1* and *DRD4* genotype groups in second block (diagnosis included in the ADHD + HC group), *GIT1 × DRD4* in third block.

The results of the post hoc analyses of the interaction effects are summarized in Table 3. Compared with the *GIT1* C/T + DRD4 others group, the *GIT1* C/T + *DRD4* 4R/4R group had significantly more commission errors (*p *<* *.001) and greater response time variability (*p *=* *.003) in the ADHD + HC group, as well as more omission errors and commission errors and greater response time variability (*p *=* *.001, *p *<* *.001, and *p *<* *.001, respectively) in the ADHD group (Figure 1). However, these associations were not significant when the *GIT1* C/C group was analyzed separately.

**Table 3:** Effects of *DRD4* gene variant on CPT according to *GIT1* genotype group

Variables	*GIT1*, C/C				*GIT1*, C/T			
	*DRD4* 4R/4R	*DRD4* others	*p*‐value	Cohen's *d*	*DRD4* 4R/4R	*DRD4* others	*p*‐value	Cohen's *d*
CPT scores, mean (SD)								
ADHD + HC								
Omission errors	63.1 (20.4)	61.2 (19.1)	.417	0.10	63.5 (18.7)	52.9 (14.2)	.037	0.64
Commission errors	63.1 (18.7)	61.6 (18.6)	.478	0.08	67.9 (21.5)	49.1 (8.0)	<.001	1.16
Response time variability	59.5 (17.9)	59.1 (17.6)	.829	0.02	65.2 (15.7)	51.3 (11.7)	.003	1.00
ADHD								
Omission errors	67.4 (21.0)	64.4 (20.1)	.289	0.16	65.2 (19.3)	49.8 (6.6)	.001	1.07
Commission errors	65.5 (19.1)	64.8 (19.3)	.792	0.04	71.2 (21.7)	49.3 (9.0)	<.001	1.32
RTV	63.1 (18.1)	62.4 (17.7)	.773	0.04	66.7 (15.9)	50.2 (10.0)	<.001	1.24
HC								
Omission errors	53.9 (15.6)	52.1 (12.2)	.572	0.13	54.8 (13.4)	62.5 (26.2)	.905	0.37
Commission errors	59.1 (16.8)	52.4 (12.6)	.072	0.38	51.0 (9.8)	48.5 (4.4)	.730	0.32
Response time variability	51.7 (15.2)	49.3 (13.1)	.451	0.17	57.4 (12.7)	54.0 (17.4)	.730	0.19

CPT, continuous performance test; SD, standard deviation; ADHD, attention deficit/hyperactivity disorder; HC, healthy control; ADHD + HC, ADHD and HC groups combined.

There were no significant results in the three‐way interaction analysis of diagnosis, *GIT1* genotype and *DRD4* genotype (Table 4).

**Table 4:** The three‐way interaction analysis of diagnosis, *GIT1* and *DRD4* genotype

Variables	Diagnosis		*GIT1*		*DRD4*		Diagnosis × *GIT1*		Diagnosis × *DRD4*		*GIT1 *×* *DRD4^a^		Diagnosis × *GIT1 *×* DRD4* a		Cohen's f^2^
	β (95% CI)	*p*‐value	β (95% CI)	*p*‐value	β (95% CI)	*p*‐value	β (95% CI)	*p*‐value	β (95% CI)	*p*‐value	β (95% CI)	*p*‐value	β (95% CI)	*p*‐value	
CPT scores, mean (SD)															
Omission errors	12.75 (−8.60, 34.10)	.241	39.71 (9.78, 69.64)	.009	38.51 (2.72, 74.30)	.035	−25.08 (−47.69, −2.46)	.030	−23.10 (−51.06, 4.85)	.105	−34.32 (−72.38, 3.75)	.077	21.91 (−7.64, 51.45)	.146	0.11
Commission errors	−0.75 (−21.29, 19.79)	.943	27.18 (−1.62, 55.98)	.064	41.39 (6.94, 75.83)	.019	−11.66 (−33.42, 10.10)	.293	−19.44 (−46.35, 7.46)	.156	−45.73 (−82.36, −9.06)	.015	24.48 (−3.96, 52.91)	.091	0.10
Response time variability	4.33 (−14.60, 23.27)	.653	29.72 (3.17, 56.27)	.028	30.23 (−1.52, 61.98)	.062	−17.46 (−37.52, 2.60)	.088	−13.66 (−38.47, 11.14)	.279	−31.21 (−64.98, 2.56)	.070	15.35 (−10.86, 41.56)	.250	0.12

CPT, continuous performance test; ADHD, attention deficit/hyperactivity disorder; HC, healthy control; ADHD + HC, ADHD and HC groups combined: CI, confidence interval; SD, standard deviation.

a Diagnosis, *GIT1* and *DRD4* genotype groups in first block; Diagnosis × *GIT1,* Diagnosis × *DRD4*, and *GIT1* × *DRD4* in second block; Diagnosis × *GIT1 × DRD4* in third block.

## 4 DISCUSSION

To our knowledge, this study is the first to investigate the interaction effects of *GIT1* rs550818 and *DRD4* 48 bp VNTR gene variants on CPT variables such as commission errors and response time variability, which are well‐established neurocognitive endophenotypes of ADHD (Kebir & Joober, 2011). This study found significant effects of *GIT1, DRD4*, and *GIT *× *DRD4* on commission errors in the ADHD group, but not in the HC group. Furthermore, post hoc analyses revealed that the *DRD4* variants were significantly associated with CPT variables in the *GIT1* C/T group but not in the *GIT1* C/C group. These results agree with those of a previous study that found that the C/T genotype is significantly associated with susceptibility to ADHD (Won et al., 2011). The diagnosis × *GIT* × *DRD4* was not significant, but due to the small sample size of this study, it would be difficult to draw a definite conclusion without further studies with larger sample sizes.

In the ADHD group, the *GIT1* C/T + *DRD4* 4R/4R group had more omission and commission errors and higher response time variability scores than the *GIT1* C/T + *DRD4* others group. These results are not in line with those of a previous study which found that the homozygosity of the 4R allele at *DRD4* is associated with fewer commission errors and less response time variability in a Korean sample of ADHD patients (Kim et al., 2009). A majority of research on *DRD4* has investigated the 7R allele, which is rare in Asian populations, including Koreans (Lichter et al., 1993). As the risk allele of this *DRD4* variant in Koreans is largely unknown (the 4R allele has been found to be a protective allele, and no study has found the risk allele yet), the results of studies comparing the effects of *DRD4* 4R/4R and other genotypes may differ according to the frequencies of each allele included in the other genotype groups (e.g., 2R, 7R, etc.).

After the first *GIT1* study by Won et al. (2011), subsequent studies have not been able to replicate the association between *GIT1* rs550818 and ADHD. For example, a Brazilian case–control study and a meta‐analysis both failed to find a significant relationship between *GIT1* rs550818 and ADHD (Klein et al., 2015; Salatino‐Oliveira et al., 2012); however, these discrepancies may be due, at least in part, to differences in the genetic backgrounds of the participants. The allele frequencies in these two replication studies (Klein et al., 2015; Salatino‐Oliveira et al., 2012) were consistent with those observed in the European population (minor allele frequency [MAF] = 0.27) but markedly different from the MAF of 0.06–0.09 reported by Won et al. (2011). Another explanation may be the lack of consideration for the interactions of *GIT1* variants with other genetic polymorphisms. As ADHD is thought to be a polygenic disorder, it has been proposed that interaction analyses would be useful to better understand the genetic influence of this disorder (Gabriela et al., 2009). The present findings suggest that this *GIT1* variant is associated with the pathophysiology of ADHD via its interaction with the dopamine system, specifically through with this *DRD4* variant. Thus, further studies investigating the role of *GIT1* variants in ADHD should consider the multifaceted interaction effects of dopamine‐related genes; for example, the interaction between multiple gene variants such as *DRD4* and dopamine transporter 1 (*DAT1*) or interactions with other catecholamine‐related genes.

Of note, this study is the first to report a significant effect of a *GIT1* variant on CPT variables in patients with ADHD. Won et al. (2011) showed that *GIT1* −/− mice exhibit ADHD‐like phenotypes, including hyperactive behavior and learning and memory impairments. However, studies that used the Sustained Attention Dots (SAD) task, digit span task, flanker task, Sustained Attention to Response Task (SART), delay discounting task, and trail‐making task to investigate the behavioral phenotype of *GIT1* in humans have all produced negative results (Klein et al., 2015; Salatino‐Oliveira et al., 2012; Won et al., 2011). The CPT is one of the most common neuropsychological tests used to assess sustained attention, inhibitory control, and attentional regulation in patients with ADHD (Castellanos et al., 2005; Sonuga‐Barke & Castellanos, 2007) and the variables of this test have been proposed as a promising endophenotype for ADHD (Kollins et al., 2008). The discrepancy between the present results and those of previous studies may be due to the reasons described above; namely, differences in the genetic backgrounds of the participants and a lack of consideration for gene–gene interactions.

Previous studies have indicated that *GIT*1 rs550818 is a functional SNP; the minor allele confers a reduction in the expression levels of *GIT1* (Klein et al., 2015; Won et al., 2011). GIT1 is a ubiquitous multidomain protein involved in diverse cellular processes and acts as a G protein‐coupled receptor kinase interacting protein that contains an adenosine diphosphate ribosylation factor GTPase‐activating protein domain (Hoefen & Berk, 2006). As mentioned above, the overexpression of GIT1 disrupts the internalization of numerous G protein‐coupled receptors (Claing et al., 2000; Hoefen & Berk, 2006; Premont et al., 1998), including dopamine receptors; this may be the mechanism underlying the *GIT1 *×* DRD4* effects on impulsivity and response time variability in ADHD patients observed in this study. Intracellular signal transduction systems are regulated by different G proteins according to the type of dopamine receptor; dopamine D1 and D5 receptors are coupled with G protein G_αs_ and activate adenylyl cyclase, whereas dopamine D2, D3, and D4 receptors are coupled with G protein G_αi_ and inhibit adenylyl cyclase (Wu, Xiao, Sun, Zou, & Zhu, 2012).

In this study, there were no significant differences regarding the distributions of the genotype or allele frequencies of the *GIT1* and *DRD4* variants between the ADHD and HC groups, in contrast to other study results. Because the genetic effects of a single SNP in ADHD populations are weak and large sample sizes may be needed to detect significant differences, the negative results of this study may be due to the small sample size. One limitation of case–control studies is proneness to population stratification. However, the Korean population has a highly homogeneous genetic background and, thus, the possibility of population stratification is unlikely. It is also possible that gene–gene or gene–environment interactions were the cause of the present discrepancies with other genetic association studies. In terms of *DRD4*, most case–control studies of the Korean population have produced negative results and consisted of small sample sizes (Ji, Paik, Park, & Lim, 2013). Thus, GWAS and/or meta‐analysis studies targeted for Asian populations will be required to determine the nature of the relationships among *GIT1*,* DRD4,* and ADHD.

This study has several limitations that should be noted. First, the sample size was relatively small for genetic analyses, particularly for the *GIT1* C/T subgroup in the HC group (*n* = 9); this may have negatively affected the statistical power of the results. There were significant differences in age and gender between the ADHD and HC groups but we included these as covariates to compensate for this limitation. Additionally, all subtypes of ADHD were included in this study because the sample size was too small to perform independent analyses according to subtype. ADHD patients may exhibit differential CPT profiles according to subtype; further research using a more behaviorally homogeneous population is warranted. Furthermore, this study recruited only Korean participants, an ethnically homogenous group, which limits the generalizability of the results to other ethnic groups. Replication studies using participants with various genetic backgrounds are required. The *GIT* effect on ADHD seems to occur only in Koreans so far. Further studies exploring if this effect is due to something in the Korean genetic background or due to the low number of individuals with the minor allele are warranted. The genotypes of the *DRD4* variant were divided into two groups without consideration for the functionality of these alleles. The *DRD4* others group was a heterogeneous group; classifying the 2R–7R alleles into a single group may have obscured the effects of each allele. Finally, only one variant for each of the *GIT1* and *DRD4* genes was analyzed in this study.

## 5 CONCLUSIONS

The present results suggest that impulsivity in ADHD patients was influenced by the interaction effects of the *GIT1* and *DRD4* gene variants. Further studies that utilize larger sample sizes and multiple gene variants are needed to clarify and confirm the present findings.
