# Supplementary material for: Interaction effects of GIT1 and DRD4 gene variants on continuous performance test variables in patients with ADHD
Source: Brain Behav. 2017 Aug 1;7(9):e00785. doi: 10.1002/brb3.785 (PMC5607549; doi:10.1002/brb3.785)
Supplement: Supplementary file 1 [file BRB3-7-e00785-s001.docx]

**Supplementary Table S1**. Allelic and genotypic distributions of the *GIT1*and *DRD4* gene variants

| Gene variant | ADHD (n = 255), N (%) | HC (n = 98), N (%) | p-value |
| --- | --- | --- | --- |
| *GIT1* rs5508181 |  |  |  |
| Genotype |  |  | 0.157 |
| CC | 217 (85.1) | 89 (90.8) |  |
| CT | 38 (14.9) | 9 (9.2) |  |
| Allele |  |  |  |
| C | 472 (92.5) | 187 (95.4) |  |
| T | 38 (7.5) | 9 (4.6) |  |
| *DRD4* 48-bp VNTR |  |  |  |
| Genotype |  |  | 0.432 |
| 4R/4R | 147 (57.6) | 61 (62.2) |  |
| Others | 108 (42.4) | 37 (37.8) |  |
| Allele |  |  |  |
| 2R | 90 (17.6) | 28 (14.3) |  |
| 3R | 4 (0.8) | 3 (1.5) |  |
| 4R | 390 (76.5) | 153 (78.1) |  |
| 5R | 22 (4.3) | 8 (4.1) |  |
| 6R | 3 (0.6) | 3 (1.5) |  |
| 7R | 1 (0.2) | 1 (0.5) |  |

Abbreviations: ADHD, attention-deficit/hyperactivity disorder; HC, healthy control; VNTR, variable number tandem repeat; R, repeat

**Supplementary Table S2.** Genotype combinations in ADHD and HC groups

| Gene variant | ADHD (n = 255) | | HC (n = 98) | |
| --- | --- | --- | --- | --- |
|  | GIT1, C/C  (n = 217) | GIT1, C/T + T/T  (n = 38) | GIT1, C/C  (n = 89) | GIT1, C/T + T/T  (n = 9) |
| *DRD4* 48-bp VNTR, N %) |  |  |  |  |
| 4R/4R | 121 (55.8) | 26 (68.4) | 56 (62.9) | 5 (55.6) |
| Others | 96 (44.2) | 12 (31.6) | 33 (37.1) | 4 (44.4) |

Abbreviations: ADHD, attention-deficit/hyperactivity disorder; HC, healthy control; VNTR, variable number tandem repeat; R, repeat.
